# Assessment of Preference Behavior of Layer Hens under Different Light Colors and Temperature Environments in Long-Time Footage Using a Computer Vision System

**DOI:** 10.3390/ani13152426

**Published:** 2023-07-27

**Authors:** Vanessa Kodaira, Allan Lincoln Rodrigues Siriani, Henry Ponti Medeiros, Daniella Jorge De Moura, Danilo Florentino Pereira

**Affiliations:** 1 Graduate Program in Agricultural Engineering, Faculty of Agricultural Engineering, Campinas State University, Campinas 13083-875, SP, Brazil; vkodaira@gmail.com (V.K.); danilo.florentino@unesp.br (D.F.P.); 2 Graduate Program in Agribusiness and Development, School of Science and Engineering, São Paulo State University, Tupã 17602-496, SP, Brazil; allan.siriani@unesp.br; 3 Department of Agricultural and Biological Engineering, University of Florida, Gainesville, FL 32611, USA; hmedeiros@ufl.edu; 4 Department of Management, Development and Technology, School of Science and Engineering, São Paulo State University, Tupã 17602-496, SP, Brazil

**Keywords:** environmental stress, precision poultry farming, welfare, YOLO

## Abstract

**Simple Summary:**

Preference behavior can be an important indicator of animal welfare. The effects of different wavelengths of light on laying hens are not completely known. We performed a preference test with few laying hens with three different light sources (green, red, and white) in three thermal environmental conditions (cold, comfort and heat stress). We recorded video footage during the entire period of the experiment and automatically estimated the permanence of the birds in each room of the Environmental Preference Chamber using YOLO v4. The results showed that birds prefer white and red light under thermal comfort conditions and do not show preference under heat stress conditions.

**Abstract:**

As for all birds, the behavior of chickens is largely determined by environmental conditions. In many production systems, light intensity is low and red feather strains have low contrast with the background, making it impossible to use conventional image segmentation techniques. On the other hand, studies of chicken behavior, even when using video camera resources, depend on human vision to extract the information of interest; and in this case, reduced samples are observed, due to the high cost of time and energy. Our work combined the use of advanced object detection techniques using YOLO v4 architecture to locate chickens in low-quality videos, and we automatically extracted information on the location of birds in more than 648 h of footage. We develop an automated system that allows the chickens to transition among three environments with different illuminations equipped with video cameras to monitor the presence of birds in each compartment, and we automatically count the number of birds in each compartment and determine their preference. Our chicken detection algorithm shows a mean average precision of 99.9%, and a manual inspection of the results showed an accuracy of 98.8%. Behavioral analysis results based on bird unrest index and permanence time indicate that chickens tend to prefer white light and disfavor green light, except in the presence of heat stress when no clear preference can be observed. This study demonstrates the potential of using computer vision techniques with low-resolution, low-cost cameras to monitor chickens in low-light conditions.

## 1. Introduction

Illumination is a key environmental factor impacting egg production, as it alters the bird’s physiology, behavior, and well-being [1,2]. Light intensity and color are two important parameters that affect bird behavior [3]. The wavelength defines the colors perceived by the animals, impacting their behavior and consequently their response to the rearing environment, whether in stress relief or in improving the immune response [4]. The avian eye has photoreceptors in the retinal cone that are sensitive to a broader portion of the light spectrum than humans, allowing birds to perceive wavelengths between 350 nm and 700 nm [5]. This wavelength range encompasses ultraviolet rays [6], which increase productivity and encourage various desirable behaviors [7,8].

White light is commonly used for rearing laying hens [9,10] because it has a balanced distribution of the light spectrum in all wavelengths. Several studies indicate that short-wavelength light (green and blue) stimulates bird growth, improves animal welfare [11,12,13,14], and leads to higher quality eggs with increased weight, thickness, and shell strength [9,15]. Long-wavelength light (red and orange) increases reproductive hormone levels, favoring the development of sexual organs, influencing the age of sexual maturation of chickens, and improving productive performance [11,16,17,18]. On the other hand, these wavelengths also promote unwanted behaviors, such as aggressive pecking and cannibalism [9,12].

It is possible to monitor the behavior of chickens in different environmental conditions using video cameras. These behaviors can be analyzed either manually or automatically, using computer vision techniques and deep learning models for object detection [19]. Traditionally, the application of automated object detection techniques required substantial expertise in the design of visual feature extraction methods and feature similarity evaluation strategies. Deep learning methods changed that paradigm. It is now possible to apply computational models composed of multiple layers of abstraction that can directly represent the relationships between the inputs and outputs of the problem to be solved based on observed data. Such models are especially effective for problems for which there are no solutions based on traditional artificial intelligence and machine learning methods [20].

New tools based on machine vision are being developed with the aim of extracting information on the behavior of birds automatically in recorded videos. Sudebi et al. [21] developed and evaluated a model based on YOLO v5 for detecting pecking behavior in cage-free chickens and used 1924 images, 1300 for training, 324 for validation, and 300 for testing and obtained an accuracy of approximately 88%. Yang et al. [22] used YOLO v5 to classify six broiler behaviors in 9600 images for training and 2400 images for validation, obtaining global accuracy above 95%. Guo et al. [23] found accuracy above 96% in a brown chicken detection model in 720 images with different numbers of chickens previously selected. Pu et al. [24] used deep learning models to classify the behavior of birds in feeders, using color and depth images (i.e., a Kinect camera), obtaining a classification accuracy of 99%. Lin et al. [25] implemented a system to detect heat stress in birds, monitoring bird activity, temperature, and relative humidity. Wang et al. [26] designed a bird behavior classifier, achieving an accuracy of 95% in the detection of six classes of behaviors. An image-based system for detecting sick birds was developed by Zhuang and Zhang [27] and showed an accuracy of 99.7%. The aforementioned studies, as well as the vast majority of research using machine vision to detect chickens and analyze the behavior of these animals, consist of proofs of concept that test the effectiveness of the models in small image samples.

Commercial laying aviaries normally operate with low illumination levels. Red hues, more common in cage-free production systems, provide low contrast between the bird and the background of the image in these low-light conditions, making it difficult to recognize the birds using computer vision techniques. The bird detection model developed by Siriani et al. [28], based on the YOLO v4 architecture, effectively solved this problem, detecting birds in low-contrast images with an accuracy of 99.9%.

Our hypothesis is that laying hens have preferences for specific wavelength of illumination and that the ambient temperature can affect this preference behavior. Thus, we use the bird detection system proposed by Siriani et al. [28] to analyze footage of an experiment in a climatic chamber and evaluate the preference behavior and activity level of chickens for environments with different illumination sources (white, green, and red), under three environmental conditions (thermoneutral, cold, and hot).

## 2. Materials and Methods

### 2.1. Experimental Design

**Figure 1:**
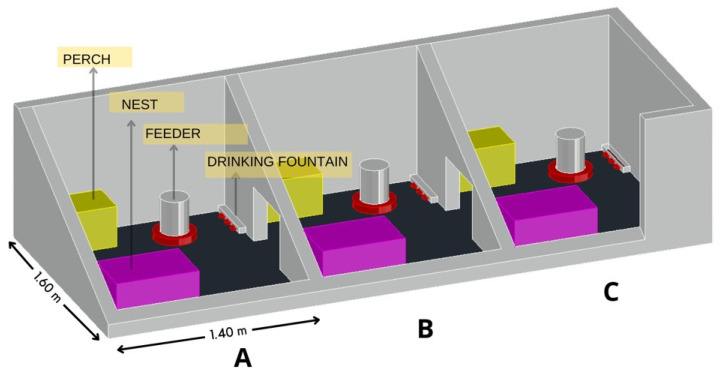
Environmental Preference Chamber (EPC) layout. The EPC comprises three identical compartments: (**A**–**C**).

Six H&N Brown Nick layer hens, 35-weeks old at the beginning of the pilot experiment, were housed in an Environmental Preference Chamber (EPC) at the Faculty of Agricultural Engineering of the Campinas State University. The EPC comprises three mutually insulated compartments, measuring 1.6 m × 1.4 m × 3.0 m (L × W × H), enriched with a wood shavings bed (5 cm deep), a perch (60 cm length and 30 cm height), and a nest. The compartments are interconnected through automatic doors that allow the birds to move freely and choose their preferred environment, as shown in Figure 1. Before we initiated the experiments, the hens went through an adaptation period of seven days to gain familiarity with the EPC and learn to access its compartments.

After the training period, the birds were subjected to treatments with different light colors and ambient temperature, according to the experimental design shown in Table 1. Each compartment was illuminated using an LED light source of different color (white 3000 K, red, and green) and the same luminance of ~5 lux. The photoperiod was 16 h of light and 8 h of complete darkness. Bird preference behavior was monitored for three temperatures: 35 °C (Hot), 24 °C (Thermoneutral), and 17 °C (Cold). In each experiment, the temperature setting in the EPC was the same for all the compartments, so that the birds’ preference for the location was determined solely by the lighting conditions in that environment. The combined light and temperature treatments were rotated in the EPC compartments, with each treatment lasting two days, according to the protocol proposed by Ma et al. [29]. Each experiment listed in Table 1 was repeated three times, for a total of 54 days of experimentation.

### 2.2. Preference Behavior Monitoring

The behavior of the birds was recorded using low-cost video cameras with a resolution of 702 × 480 pixels and a capture rate of 30 fps (CCD sensor 1/4″, 2.6 mm lens, Intelbras^®^ iM5, São José, SC, Brazil). We installed one ceiling-mounted camera at the center of each EPC compartment with the camera optical axis perpendicular to the floor of the compartment so that the field of view of the camera covers the entire floor area of its respective compartment.

Although videos of the three compartments were recorded uninterruptedly for the duration of the experiments, we select two periods of two hours per day to monitor the behavior of the birds. As first observed by Bizeray et al. [30] and more recently by Grebey et al. [31], monitoring bird behavior at different time periods helps to compensate for natural behavior variations throughout the day. Hence, to minimize the impact of observation time on the behavior of the birds, we monitored one interval in the morning (9:30–11:30) and one in the afternoon (15:00–17:00). This resulted in a total of 648 h of videos across the three EPC compartments. Given the high correlation among subsequent video frames, we down-sample the observed videos to 15 fps. Thus, our evaluation dataset comprises approximately 35 million video frames.

### 2.3. Bird Detection Workflow

**Figure 2:**
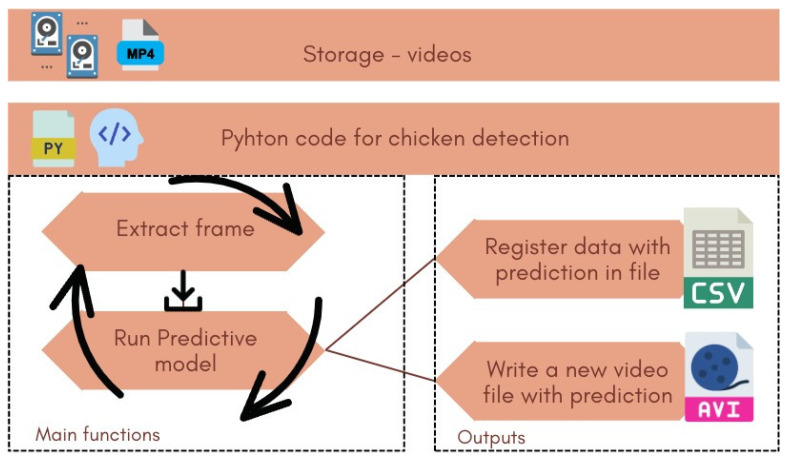
Model execution diagram and corresponding output files.

The workflow for our automatic chicken detection system is shown in Figure 2. A hard disk drive was used to store the videos in MP4 format. We developed a Python script to scan these video files and arrange them in a queue for further processing. A separate script corresponding to the bird detection model identifies the boundaries of birds as bounding boxes and calculates their centroids. After detection, the number of centroids present in each frame is stored in a structured CSV file, which also associates the results with the treatment information and respective EPC compartment.

#### 2.3.1. Computer Vision Model Design

**Figure 3:**
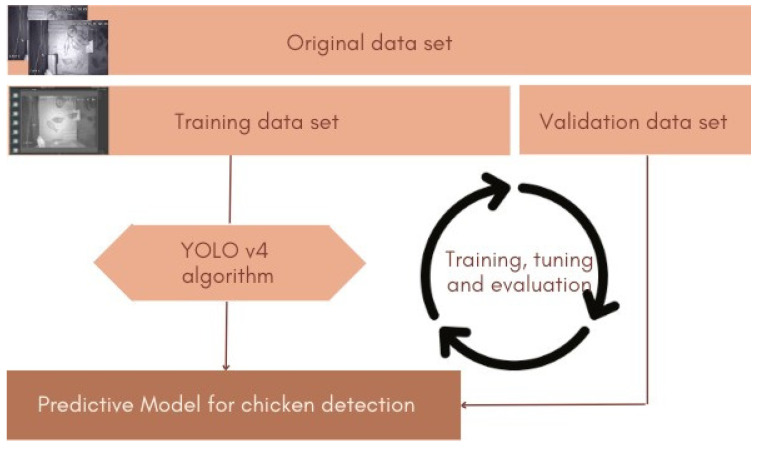
Diagram of the bird detection methodology used to build the bird behavioral analysis model.

Our bird detection model is based on the YOLO (You Only Look Once) single-stage framework for object classification and detection [32,33,34]. Unlike two-stage methods, in which image pixels are first coarsely segmented into regions of interest that are processed individually, the YOLO framework analyzes all the image pixels in a single step, which makes the model substantially faster than its two-stage counterparts. YOLO v4 [35] is a recent variant of the YOLO framework. It detects objects of different sizes in low-resolution images with an accuracy comparable to that of most state-of-the-art object detectors in a fraction of the time required by these methods. In this work, we apply the methodology for bird detection using YOLO v4 described by Siriani et al. [28], which follows the steps shown in Figure 3.

The number of frames used to train our model was defined using the sample size estimation strategy proposed by Agranonik and Hirakata [36], which is calculated as:

(1) $$n=\frac{p\left(1-p\right)Z^{2}N}{\varepsilon^{2}(N-1)+Z^{2}\;p(1-p)}$$

where *n* is the sample size, p is the expected proportion of frames containing birds, Z is the normal distribution value for a given confidence level, N is the population size, and ε is the desired margin of error. In this work, we set the confidence level to 99%, which corresponds to Z=2.576 and a margin of error ε=4%. Since no prior information is available regarding the distribution of the birds, we conservatively set p=0.5. Thus, for a population size of N=35 M, which represents the total number of video frames acquired in our experiments, we obtain a sample size of n=1041 frames.

**Figure 4:**
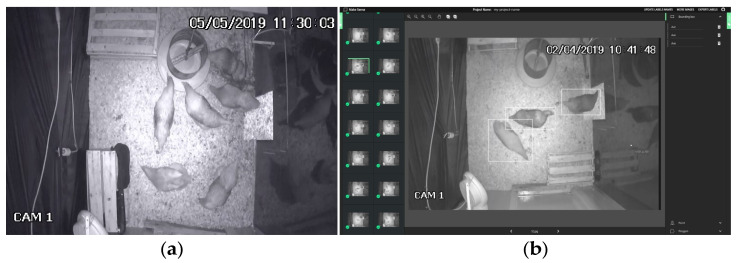
(**a**) Sample image illustrating the training set. (**b**) Manual annotation of chickens presents in the images that compose the training dataset.

We selected 1041 random frames containing birds to compose our training dataset. Our validation dataset consisted of 200 additional frames selected at random from the original videos. Figure 4a shows one of the original frames from our training set. For both datasets, we use the MakeSense tool (www.makesense.ai, accessed on 10 March 2021) to delimit a rectangular region representing the space occupied by birds in the video frames and to manually annotate the bounding boxes corresponding to each bird, as illustrated in Figure 4b.

Our object detection system is based on the Python implementation of the YOLO v4 model provided in the Darknet framework [37]. We modify the model architecture to predict only one object category and train it using the dataset described above. We configure our model to generate 16 subdivision per image, and train it using a batch size of 16, weight decay of 5 × 10^−4^, and a multi-step learning rate scheduling policy, with an initial learning rate of 1.3 × 10^−3^, which is divided by 10 after 8000 and 9000 iterations. The performance of this model applied to chicken detection within the EPC compartments is discussed in Section 3.1.

#### 2.3.2. Validation of the Bird Detection Model

We assess the effectiveness of the YOLO v4 model based on its mean average precision (mAP) on the 200 frames of our validation set. The mAP is a widely used metric to assess the accuracy of detection models based on the YOLO network [38]. It is calculated as:

(2) $$mAP=\frac{1}{n}\sum_{i=1}^{n}{AP}_{i}$$

where $AP_{i}$ is the model precision for the i-th object category and n is the number of categories. Since our application contains only one category, n=1 and $mAP={AP}_{1}$, which is simply the average percentage of detected birds on the validation set.

**Figure 5:**
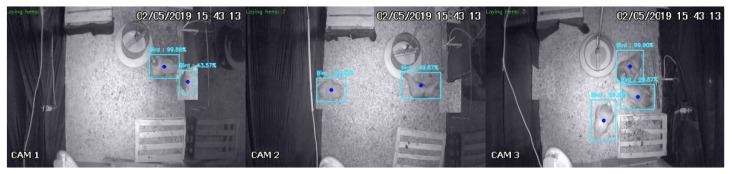
Sample frames simultaneously acquired from the three EPC compartments to manually validate the YOLO v4 detection model. The left and center frames illustrate the scenario where one bird can be partially observed in two compartments simultaneously.

We further validate the model by manually inspecting an additional 299 randomly selected scenes. Each scene comprises one video frame from each EPC compartment, for a total of 897 synchronized frames. Since the video frames are acquired simultaneously, the same bird may occasionally be partially observed in two cameras, as illustrated in Figure 5. In these scenarios, we consider the bird to be present in the compartment where its head is located and disregard the corresponding detection from the frame where the remainder of its body is observed. The manually annotated bounding boxes are considered the gold standard for comparison with the results obtained by the YOLO v4 network for the same video frames. We compute the number of correct and incorrect detections and summarize them using a confusion matrix.

### 2.4. Preference Behavior Analysis

Upon validation, we used the YOLO v4 model to detect chickens in the 648 h of videos acquired in the EPC compartments. These detections were used to estimate the mobility of the birds according to two criteria: unrest index and permanence time.

#### 2.4.1. Unrest Index Computation

Del Valle et al. [39] developed an index for the analysis of the agitation of chickens observed in subsequent video frames, which is calculated as:

(3) $${Unrest\;Index}_{\left(i,i-1\right)}=k.\max\left\{d\left(F_{\left(i\right)},F_{\left(i-1\right)}\right),\;d\left(F_{\left(i-1\right)},F_{\left(i\right)}\right)\;\right\}$$

where k is a proportionality factor, $F_{\left(i\right)}$ and $F_{\left(i-1\right)}$ are the sets of bounding boxes for chickens detected at frames observed at times i and i−1, respectively, and d(·,·) is the Euclidean distance between the centroids of the bounding boxes in the two frames. The proportionality factor is calculated according to the camera’s field of view. In our system, the cameras have a focal length of 2.8 mm, resulting in a field of view of 90°, and k=1. One important characteristic of the unrest index is that it does not depend on the individual identities of the chickens, which our detection algorithm is unable to determine. Thus, we use the centroids of the bounding boxes generated by our detector at each video frame to calculate the unrest index and compare its value among different treatment groups using Tukey’s test at a 5% significance level.

#### 2.4.2. Permanence Time Estimation

To further assess the mobility of the chickens, we also calculate the cumulative time spent by the birds in each compartment, according to:

(4) $$T_{L}=\sum_{j=1}^{m}t_{j}$$

where $T_{L}$ is the total time of permanence of the birds in light treatment L, *t_j_* is the time of permanence of the j-th bird, and *m* is the number of birds observed in the compartment during all the recordings of one experimental scenario described in Table 1.

Since our algorithm is unable to differentiate individual birds, dwell time was approximated by summing the total number of chickens identified in each frame, divided by the capture rate of 30 frames per second. The result is the total time in seconds that the chickens spend in the video. Given the high frame rate of the videos collected in our experiments and the high precision of the bird detection model, we expect this approach to accurately estimate permanence time. Differences between the total length of stay of the birds were tested among the different light and temperature treatments using one-way ANOVA and later compared using Tukey’s mean test at a significance level of 5%.

## 3. Results

### 3.1. Detection Model Validation

**Figure 6:**
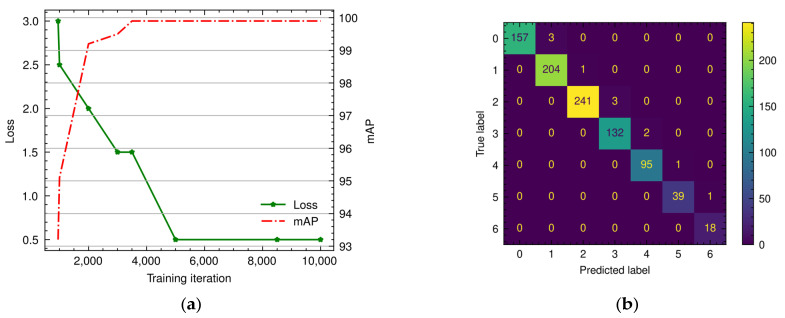
Manual performance validation of the YOLO v4 detection model. (**a**) Validation loss and mAP as a function of training iterations. (**b**) Confusion matrix for the 299 manually validated simultaneous scenes.

Figure 6a summarizes the performance of our detection model as a function of the training iterations on the 200 images composing our validation set. After approximately 5000 iterations, the loss value stabilizes at 0.5, and the mAP plateaus at ~99.9% after 3500 iterations. Although we cannot rule out model overfitting considering the similarity of the images in the training and validations sets, in this experimental environment this behavior is acceptable because all the frames used for the analysis were obtained under virtually identical conditions.

Figure 6b shows the confusion matrix for the visually inspected video frames used to manually validate the model. As expected, between zero and six birds were present in each scene, and the model predictions reflect this with an accuracy of 98.8%. As explained in Section 3.1, we accounted for chickens observed in two frames in the generation of our ground truth annotations. However, that does not preclude the automatic algorithm from detecting both parts of the chicken in both chambers. This would result in the system occasionally overestimating the number of birds in one of the chambers by one. Figure 6b indeed seems to support that hypothesis, as all of the mistakes made by the algorithm indeed represent the prediction of one additional bird in one of the frames. However, this problem only affected 1.23% of the frames under consideration (11 out of 897) and can thus be safely disregarded. Overall, these results indicate that it is possible to rely on the automatic detection of chickens for the behavioral analysis carried out in the next step.

### 3.2. Unrest Index Analysis

**Figure 7:**
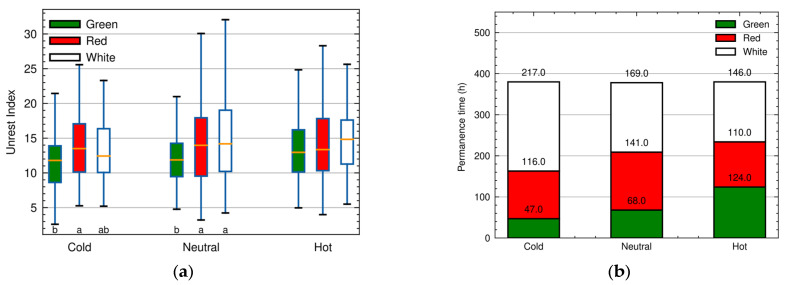
(**a**) Unrest index values for each combined temperature and illumination condition and Tukey statistical test result—the letters indicate significant difference (*p* < 0.05) in Tukey’s test between illumination treatments for each temperature condition. (**b**) Cumulative permanence time in hours for each combined light and temperature treatment.

Figure 7a shows the unrest index values for each treatment calculated according to Equation (3). The figure also shows the result of Tukey’s statistical analysis. It is possible to observe that under heat stress conditions the birds do not express significant differences in movement between the light treatments, while in the thermoneutral and cold conditions, the birds move less under green lighting. There were no differences in the movement of the birds between the temperature treatments when the light treatment was fixed. ANOVA did not indicate differences in bird movement between different temperatures, but it did indicate differences for lighting, with birds moving less under green light.

### 3.3. Bird Permanence Time

Figure 7b shows the cumulative permanence time of the birds for each treatment computed using Equation (4). It can be observed that the preference of birds for light color is affected by the temperature of the environment. In the cold environment, the birds crowded more in the EPC compartment illuminated with white light, while in the hot environment they did not show a clear preference for any light color. The ANOVA analysis showed that there is an interaction between light and temperature treatments, which can be confirmed in Table 2, which presents the differences in the average length of stay of hens in the combined treatments.

## 4. Discussion

Illumination exerts an important influence on the behavior and welfare of birds. Our work analyzed the behavior of laying hens in more than 35 million frames or 648 h of recorded videos, positioning itself as one of the first studies to use computer vision to extract knowledge about the preference of hens to different light treatments. Our measured bird detection accuracy was 99%, and we used low-quality images obtained by low-cost cameras.

Several studies have evaluated the behavior of chickens using video cameras, but analysis always depends on human observation. Mendoza et al. [40] verified the effects of UV light on the behavior of laying hens at 1 min intervals for 6 min before and 6 min after application of light treatments. Geng et al. [41] evaluated the effects of light on circadian rhythmic behavior by positioning two observers on a walkway out of the hens’ field of vision so as not to affect the birds’ normal activities. Sun et al. [42] evaluated the nesting behavior of hens in enriched cages of different sizes, in videos recorded between 6:00 and 11:00 a.m. two days a week, for 20 weeks. These works are examples of the limitation of sample sizes, and yet, they are extremely labor intensive for the observers who recorded the behavior of the animals.

The use of low-resolution images can be a viable option for environments where capturing high-quality images is not possible, either due to equipment or budget limitations. This approach can be applied in different areas of animal monitoring. One of the main advantages of using low-resolution images is the reduction in image file size, which makes storing and sharing collected data easier. This is especially useful in environments with limited bandwidth for data transfer, such as remote or hard-to-reach areas. One more benefit is the low-cost of capture equipment and the low-cost of computers for processing these data.

However, it is important to note that using low-resolution images can affect the accuracy of object detection, especially in situations where the objects to be detected are small or have complex features. Therefore, it is necessary to carefully evaluate the limitations and advantages of using low-resolution images in each specific context. In summary, the bird detection model in low-resolution images is an effective and economical solution for animal monitoring in real environments. The application of advanced image processing and machine learning techniques can maximize detection accuracy, allowing the implementation of more animal monitoring projects in different parts of the world.

The experimental results indicate that chicken have a preference for white light, followed by red, and lastly green. According to Govardovskii and Zueva [43] and Hart et al. [44], birds have four cones in their vision system and are sensitive to the light spectrum range between 350 nm and 700 nm. White light provides a strong stimulus for the perception of the environment, as it involves a broad range of the birds’ visual perception system [5,45]. This is in agreement with the studies by Lewis and Morris [6] and Gunnarsson et al. [46], in which chickens preferred white light. However, Prayitno et al. [47] reports different results. In their study, the chickens preferred green light instead of white or red.

In all the scenarios under consideration, green light was the least preferred by the birds. These results are similar to those presented by Rierson [48], who also observed the preference for white light, followed by red, while green was the least preferred by broilers. Prayitno et al. [47] reports no difference in preference between red and white, but under red light the birds showed a more aggressive behavior in comparison to white, green, and blue light. Analyzing the unrest index (Figure 7a), we observed that the birds were indeed more agitated in environments with red light, except in the presence of heat stress, which is in agreement with the results obtained by Sultana et al. [49], Hesham et al. [50], and Khaliq et al. [51].

Thermal discomfort seems to prevail over lighting preference for chickens. In the cold environment, the birds express a clear preference for white light, concentrating in groups of three or four birds in the EPC compartment illuminated with white light. On the other hand, under heat stress, the birds were equally distributed among the light treatments in groups of two birds per compartment, on average. Red lighting has the potential to reduce stress in laying hens and reduce the occurrence of feather pecking and cannibalism [7,14,52], which may have contributed to the choice and permanence of birds. Under green light, birds tend to explore the environment less [7], which naturally occurs when birds are under heat stress. These are hypotheses that we raised about the reasons why we did not find a clear preference for a type of lighting under heat stress.

Ours study is limited to an EPC experiment with few animals, allowing assessment of the preference behavior of few chickens. However, we were able to accurately assess flock preference in long footage, using a computer vision system based on deep learning, allowing us to confirm our initial hypothesis. The results presented correspond only to these chickens and to the conditions described in the experiment and can subsidize other studies of preferred behavior both in the method of analysis approached and for the comparison of the results shown.

## 5. Conclusions

We present a high-performance system to automatically monitor the behavior of layer hens using computer vision techniques. We detect the birds using the YOLO v4 model with nearly perfect accuracy and use these automatic detections to determine the distribution of birds among compartments over 648 h of footage, surpassing any previous behavioral assessment experiment ever published. Our behavior preference analysis revealed that birds prefer white light, followed by red, under the experimental conditions described in this article. This study also indicates that chickens generally avoided green light and were more agitated under red illumination. However, temperature influenced the behavior of the birds more than the color of the light source. Under heat stress, the birds preferred to move away from one another, evenly splitting between the three light treatments. Under cold stress, on the other hand, the birds concentrated more on the chamber with white illumination.

This study indicates that light color may be an important environmental factor influencing the well-being and consequently the productivity of layer hens. Most importantly, this work highlights the importance of techniques to mitigate thermal stress, whose impact is significantly more prevalent than light color differences. Overall, our findings shed light on the interplay between temperature and illumination conditions as potential stressors for commercial birds. Hence, integrated management strategies for commercial layer chicken operations should be applied. We demonstrated the technical viability of using low-resolution standard security video cameras (which are consequently low-cost and for commercial use) in poor lighting conditions, in a high-performance computer vision system that allowed us to accurately study the preference behavior of chickens.

## Figures and Tables

**Table 1:** Combinations of illumination color and thermal conditions for the three EPC compartments used in our experiments. W, G, and R stand for white, green, and red illumination. Each experiment lasted two days and was repeated three times (*n* = 3).

Exp.	Thermal	Compartment
#	Env.	ABC
1		WGR
2	Hot (35 °C)	RWG
3		GRW
4		WGR
5	Neutral (24 °C)	RWG
6		GRW
7		WGR
8	Cold (17 °C)	RWG
9		GRW

**Table 2:** Average time (minutes) spent per chicken in each combined temperature and light treatment.

Light	Temperature					
	Cold		Comfort		Heat	
Green	291.3 ± 65.7	^A,B,c^	453.9 ± 93.0	^A,b^	824.1 ± 85.7	^A^
Red	670.0 ± 207.0	^b^	936.0 ± 140.0	^a^	728.0 ± 108.0	
White	1354.0 ± 165.0	^a^	1131.0 ± 127.0	^a^	974.0 ± 134.0	

Lowercase letters indicate differences between light treatments and uppercase letters indicate differences between temperature treatments in the Tukey test (*p* < 0.05).

## Data Availability

Data will be available upon request to the corresponding author.
